# Acute Pericarditis in a Patient With Poland Syndrome

**DOI:** 10.7759/cureus.97229

**Published:** 2025-11-19

**Authors:** Gautami S Patel, Rakesh Gupta

**Affiliations:** 1 Internal Medicine, Flushing Hospital Medical Center, New York, USA; 2 Cardiology, Flushing Hospital Medical Center, New York, USA

**Keywords:** absent pectoral muscles, acute pericarditis, chest pain, chest wall deformity, pleuritic chest pain, poland syndrome

## Abstract

Poland syndrome is a rare congenital anomaly characterized by unilateral absence or hypoplasia of the pectoralis major muscle. We report the case of a 32-year-old man with previously undiagnosed Poland syndrome who presented with acute, severe, tearing chest pain radiating to the back, which worsened with sitting up and improved with lying down. The cardiac physical exam was negative for murmur and friction rub. Electrocardiograms showed focal ST-segment elevations in precordial leads V2-V4, and serial troponin levels were negative. Life-threatening cardiac causes of chest pain, such as acute coronary syndrome, aortic dissection, pulmonary embolism, and pneumothorax, were ruled out by a CT (computed tomography) scan. A transthoracic echocardiogram demonstrated a trivial anterior pericardial effusion with preserved ventricular function. Three out of four criteria for acute pericarditis were met. Treatment with colchicine 0.6 mg daily and indomethacin 25 mg three times daily resulted in complete resolution of symptoms within one week. To our knowledge, there are no reported cases of pericarditis in patients with Poland syndrome. However, hypothetically altered chest wall anatomy in Poland syndrome may predispose patients to pericardial irritation leading to pericarditis, which is idiopathic in nature.

## Introduction

Poland syndrome is a rare congenital anomaly characterized by the absence of hypoplasia of the major and minor pectoral muscles, breast or nipple anomalies, hypoplasia of subcutaneous tissue, chest wall deformities with absence of ribs, pectoral and axillary alopecia, and hand anomalies, which is usually unilateral; however, it can be bilateral [1,2]. The incidence of the syndrome is reported to be between 1:10,000 and 1:100,000 [3]. While etiology remains uncertain, the hypothesis of interruption of the embryonic blood supply of the subclavian arteries during fetal development could explain the agenesis of the pectoralis muscles [4].

Poland syndrome is primarily a musculoskeletal disorder and is not commonly associated with cardiac pathology. However, due to congenital chest wall abnormalities, clinical presentations involving chest pain in these patients may pose a diagnostic challenge. Acute pericarditis, an inflammatory condition of the pericardium, is most often idiopathic or viral in origin and is typically characterized by sharp or pleuritic chest pain, which may mimic other cardiothoracic conditions [5,6].

We present a unique case of a patient with Poland syndrome, confirmed by the absence of the left pectoral major and minor muscles and ipsilateral brachydactyly and a history of syndactyly repair, who presented with tearing chest pain and was subsequently diagnosed with acute pericarditis after ruling out other cardiovascular causes of chest pain. To our knowledge, this is an uncommon presentation, and it underscores the importance of thorough diagnostic evaluation in patients with congenital thoracic anomalies who present with chest pain. 

## Case presentation

In May 2025, a 32-year-old Hispanic man presented to the emergency department of our institution with acute chest pain. He reported that the pain had started approximately 24 hours before presentation and had progressively worsened. The pain was central in location, tearing in nature, and radiated to the back. He rated it as 8/10 in intensity, significantly limiting his ability to speak and provide a detailed history. The pain was exacerbated by deep inspiration and lying down and improved with sitting up. There was no association with food intake. He denied experiencing similar symptoms in the past.

The patient also reported associated shortness of breath. He denied abdominal pain, nausea, vomiting, fever, cough, or headache. He denied recent illness or sick contact.

On examination, the patient appeared visibly uncomfortable, with persistent chest pain. Cardiac examination was negative for murmurs and pericardial friction rub. He seemed to be in fear of breathing fully, as it would increase the pain. He was vitally stable. Blood pressure was 132/85 mmHg while supine, heart rate was 80 bpm, respiratory rate was normal, and oxygen saturation was 98% on the room air. His temperature was 98.3° F.

**Figure 1:**
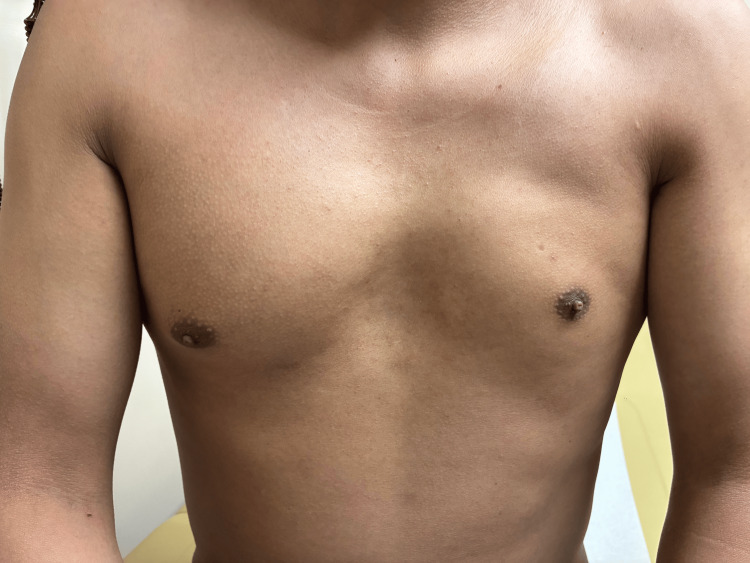
Photograph of the patient's chest showing asymmetry and the absence of pectoral muscles on the left side

**Figure 2:**
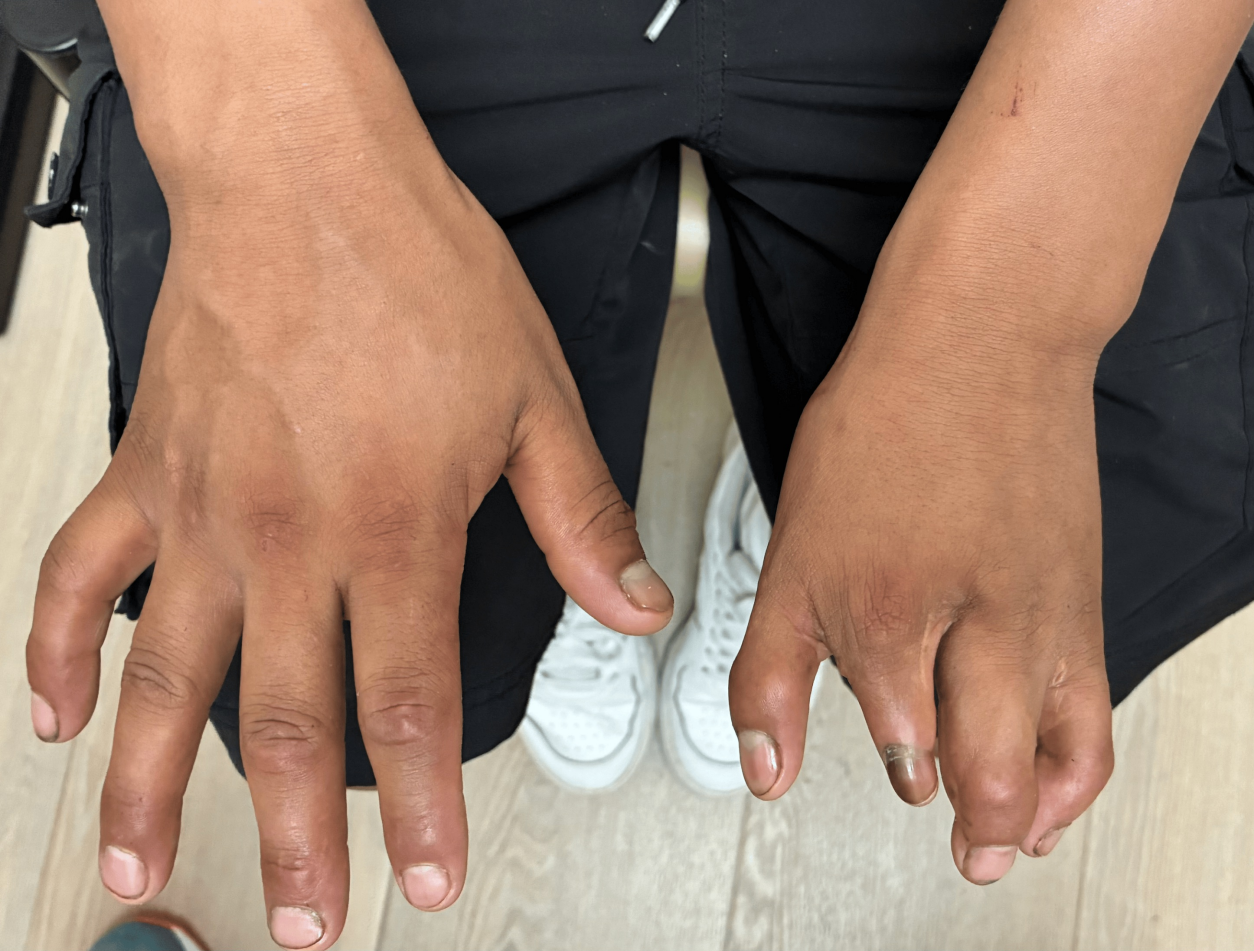
Photograph of the patient’s hand showing brachydactyly of left hand fingers with four fingers present

The patient's past medical history was notable for congenital chest wall asymmetry (see Figure 1), brachydactyly (see Figure 2), and syndactyly, with no established diagnosis of Poland syndrome. He had no history of cardiac disease or frequent hospitalizations and was not on any regular medications.

Surgical history included an appendicectomy around 15 years ago and corrective surgery for webbed fingers three years prior. He was unable to provide a picture of his hand prior to the surgery. There was no known family history of similar chest wall or limb abnormalities. There was no genetic testing done in the past as per the patient. He was a non-smoker, did not consume alcohol, and denied any substance use. He was independent in all activities of daily living and worked in construction.

The patient was admitted to the cardiac critical care unit due to severe unremitting chest pain and for close observation until acute coronary syndrome (ACS), aortic dissection, and pulmonary embolism were ruled out. 

Investigations

Laboratory Tests

Initial laboratory tests were unremarkable except for mildly elevated C-reactive protein (Table 1). Serial troponin obtained on admission and after six hours were not indicative of myocardial injury (Table 1). Urine toxicology, urine analysis and respiratory viral panel were negative. Blood culture showed no growth after five days. 

**Table 1:** Laboratory tests during the hospital stay WBC: White Blood Cell Count, RBC: Red Blood Cell Count, ESR: Erythrocyte Sedimentation Rate

Laboratory Tests	On admission	After 24 Hours	Reference range
WBC	8.4	5.2	3.8-11
RBC	5.23	4.81	4.2-5.8
Hemoglobin	15.4	14.4	13.5-17.5 g/dL
Platelet Count	249	253	130-400
Urea Nitrogen	15	28	9-20 mg/dL
Creatinine	0.5	0.8	0.7-1.3 mg/dL
Sodium	133	136	137-145 mmol/L
Potassium	3.8	4.3	3.5-5.1 mmol/L
Chloride	101	107	98-107 mmol/L
Phosphorus	4.8	5	2.5-4.5 mg/dL
Magnesium	2	2.2	1.6-2.3 mg/dL
Lactate	0.69		0.7-2.00 mmol/L
C-Reactive Protein	1.7	2.4	<1 mg/dL
ESR	3		0-15
Procalcitonin	0.05		0.03-100 ng/mL
Troponin	<0.012		<0.034 ng/mL: Normal; 0.034-0.120 ng/mL: At risk for recurrent ischemic events; >0.120 ng/mL: Recommended cut-off value for myocardial infarction

Imaging Studies

A contrast-enhanced CT scan of the chest showed no filling defects in the pulmonary vasculature. The pulmonary arteries, aorta, and other great vessels appeared grossly unremarkable. Patchy opacities were noted in the bilateral lower lobes. There was no evidence of pneumothorax, mediastinal widening, or lymphadenopathy. Osseous structures were also unremarkable.

**Figure 3:**
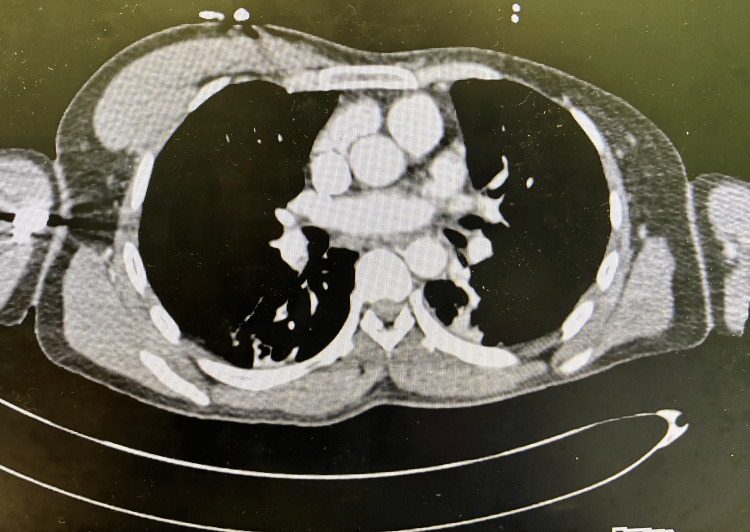
CT scan of the chest confirming the absence of left pectoral major and minor muscles

The CT scan of the chest also revealed notable asymmetry of the thoracic musculature. The left pectoralis major and pectoralis minor muscles were not visualized at the level of the sternum, consistent with congenital absence (Figure 3). These findings supported a diagnosis of Poland syndrome.

A CT angiogram of the chest and abdomen showed a normal thoracoabdominal aorta. The cardiac silhouette was normal in size, and there was no evidence of coronary artery calcification or pericardial effusion.

Cardiology Investigations

**Figure 4:**
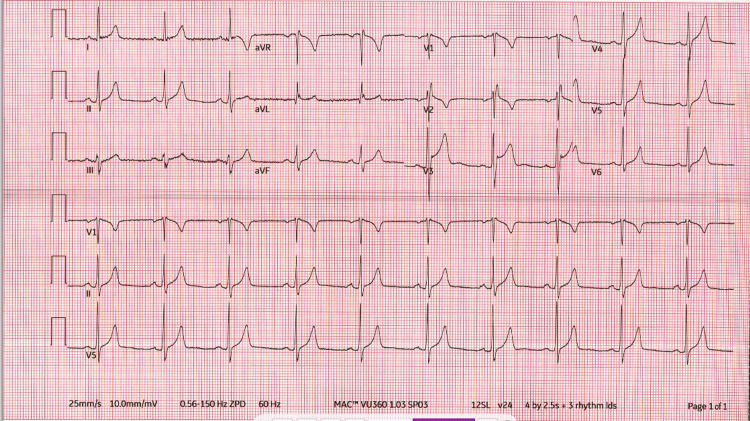
ECG showing normal sinus rhythm with localized ST elevations in precordial leads and incomplete right bundle branch block

Electrocardiogram (ECG) demonstrated normal sinus rhythm with incomplete right bundle branch block with localized ST elevations in precordial leads V2, V3, and V4 (Figure 4). 

Transthoracic echocardiography revealed a normal-sized left ventricular cavity with an ejection fraction of 55-60%. Diastolic function was preserved. All four cardiac valves were structurally and functionally normal. The aorta appeared normal in caliber and morphology. A trivial pericardial effusion was visualized anterior to the heart.

Differential diagnosis

Given the nature of the patient's chest pain, tearing, central, and radiating to the back, a broad differential diagnosis was initially considered. ACS was deemed unlikely as serial high-sensitivity troponin measurements were negative despite localized ST changes in precordial leads in the ECG, as shown in Figure 4. Aortic dissection was ruled out based on a normal CT angiogram of the chest and abdomen. Pulmonary embolism was also excluded on the basis of the same imaging study. Pneumothorax was unlikely but it was also ruled out in the same imaging study.

Bilateral lower lobe opacities noted on CT chest raised suspicion for community-acquired pneumonia, and the patient was empirically started on antibiotics, although procalcitonin remained negative. Gastroesophageal reflux disease was also considered in the differential, and pantoprazole therapy was initiated. However, the patient’s pain was not epigastric in location and not related to food intake. 

In the context of sharp, pleuritic, positional chest pain that improved with sitting up and worsened with lying down and with deep inspiration, atypical ECG changes with localized ST elevations in precordial leads, and a new small pericardial effusion on transthoracic echocardiogram, a diagnosis of acute pericarditis was made as it meets three out of four criteria of pericarditis [6]. Pericardial friction rub was not audible upon exam. The patient started on indomethacin and colchicine, resulting in marked clinical improvement.

Treatment

Following the diagnosis of acute pericarditis, the patient was initiated on colchicine 0.6 mg daily and indomethacin 25 mg three times daily [7]. The patient showed gradual improvement in chest pain with this regimen during his hospitalization.

He was also prescribed pantoprazole for gastric protection while on non-steroidal anti-inflammatory therapy. Ceftriaxone 1g every 24 hours was continued for a total of five days for empirical treatment of suspected community-acquired pneumonia based on CT findings of patchy opacities in the bilateral lower lobe, although pneumonia was ultimately ruled out based on lack of fever, white count and negative procalcitonin.

The patient remained hemodynamically stable during admission and was discharged with instructions to continue colchicine 0.6mg daily for a total of three months and indomethacin 25mg three times a day for a week followed by 25mg twice daily for the next week followed by indomethacin 25mg daily for the last week [7]. He was scheduled for outpatient follow-up with a cardiologist in three weeks after discharge to assess treatment response and monitor for recurrence.

Outcome and follow-up

The patient was presented for a three-week follow-up in the outpatient cardiology clinic after being discharged from the hospital. He reported complete resolution of chest pain, stating that symptoms subsided entirely after approximately one week of treatment. Initially, he experienced some discomfort while lying supine, which resolved with ongoing therapy.

He confirmed adherence to his prescribed medications and denied experiencing any gastrointestinal side effects. The patient was advised to return to the emergency department if similar symptoms recur. A routine cardiology follow-up was scheduled after six months. 

## Discussion

Poland syndrome is a rare congenital anomaly first described in 1841 [2], characterized by unilateral or bilateral absence or underdevelopment of the pectoralis muscle and frequently associated with ipsilateral upper limb anomalies, such as brachydactyly or syndactyly [1,2]. Some variations of the syndrome include rib defects, absence of the shoulder girdle muscle, and dextrocardia [2,8]. Classical Poland syndrome is predominantly right-sided [3], whereas our patient had left-sided absence of both pectoralis muscles and left-sided brachydactyly and syndactyly, which was repaired. Our patient does not have dextrocardia nor rib defect; a study suggests that partial agenesis of two or more ribs is needed to displace the heart towards the right side [9].

There is limited literature describing an association between Poland syndrome and pericarditis. A case of chylous pericardial effusion due to a chylous fistula in a 63-year-old man with Poland syndrome was reported due to an unusual thoracic duct anomaly in 2013 [10]. Another case was reported in Turkey in 2023, a 24-year-old woman with Poland syndrome who presented to the hospital with complaints of chest pain and dyspnea, and imaging studies revealed a large mass attached to the right atrium, which was diagnosed with primary right atrial cardiac angiosarcoma [11]. Although Poland syndrome does not predispose patients to malignancy, different pathologies can be seen in these patients due to the unknown etiology of the syndrome [11]. Another case of Poland syndrome in an 18-year-old man who presented to an orthopedic outpatient clinic with left-sided chest pain during exertion, who had a small diaphragmatic hernia and rightward deviation of the mediastinum with absence of the left pectoralis major muscle but no hand deformities [12]. One more case of a 22-year-old man with Poland syndrome presented to the emergency department with dyspnea and chest pain, who had absent breath sounds on the right side and was diagnosed with right-sided pneumothorax after imaging tests [13]. This patient was treated with tube thoracostomy [13].

Poland syndrome primarily involves musculoskeletal and cosmetic abnormalities; its anatomical variation can complicate clinical evaluations, especially in patients presenting with chest pain. In our case, a patient with undiagnosed Poland syndrome presented with acute, tearing chest pain. The absence of the left pectoral muscle initially raised concern for atypical thoracic pathology, prompting a thorough cardiovascular workup. Although no direct pathophysiological well-established link in current literature between Poland syndrome and acute pericarditis has been established, we speculate that, given this patient’s thin and small chest cavity, he may be at increased risk for pericarditis due to altered chest wall geometry, and the proximity of chest wall structures to the myocardium may make these types of patients more susceptible to irritation and inflammation. Clinicians should remain vigilant in assessing chest pain in such patients.

Acute pericarditis may result from infectious causes or non-infectious causes, although it's often idiopathic [5,6]. Management of acute pericarditis in this case followed current guidelines, with the use of nonsteroidal anti-inflammatory drugs such as Indomethacin 25mg three times a day, followed by taper for a total of three weeks and colchicine 0.6mg daily for three months [7]. The patient responded very well to treatment, with complete resolution of symptoms within one week and no adverse effects.

## Conclusions

This case highlights the diagnostic challenges posed by congenital anatomical anomalies of Poland syndrome when evaluating acute chest pain. The absence of left pectoral muscles and ipsilateral hand deformities initially raised concern for atypical thoracic pathology; however, a systematic and stepwise workup ultimately revealed acute pericarditis. Although no established pathophysiologic association exists between Poland syndrome and pericardial inflammation, altered thoracic anatomy may complicate physical examination, ECG interpretation, and imaging findings. Isolated reports describe other thoracic complications in Poland syndrome, including right atrial angiosarcoma, diaphragmatic hernia, spontaneous pneumothorax, and chylous pneumothorax in patients presenting with chest pain and dyspnea. Clinicians should maintain a high index of suspicion for common cardiac conditions in patients with congenital chest wall anomalies, carefully rule out life-threatening causes, and avoid prematurely attributing symptoms to benign etiologies. Increased awareness of Poland syndrome and its variations can facilitate timely diagnosis, appropriate management, and improved outcomes.
